# The Impact of Listening to, Reciting, or Memorizing the Quran on Physical and Mental Health of Muslims: Evidence From Systematic Review

**DOI:** 10.3389/ijph.2022.1604998

**Published:** 2022-08-31

**Authors:** Wan Nor Atikah Che Wan Mohd Rozali, Ismarulyusda Ishak, Arimi Fitri Mat Ludin, Farah Wahida Ibrahim, Nor Malia Abd Warif, Nur Aishah Che Roos

**Affiliations:** ^1^ Center for Toxicology and Health Risk, Faculty of Health Sciences, Universiti Kebangsaan Malaysia, Kuala Lumpur, Malaysia; ^2^ Center for Healthy Ageing and Wellness, Faculty of Health Sciences, Universiti Kebangsaan Malaysia, Kuala Lumpur, Malaysia; ^3^ Faculty of Medicine and Defence Health, National Defence University of Malaysia, Kuala Lumpur, Malaysia

**Keywords:** mental health, physical health, quality of life, listening, reciting, memorizing, Quran

## Abstract

**Objectives:** Listening to or memorizing the Quran has been suggested to affect the psychosocial health and well-being of Muslims. Muslims who memorized Quran have a higher quality of life (QoL) and lower anxiety and stress. Hence, this systematic review was conducted to evaluate the studies that assessed the effect of listening to, reciting, or memorizing the Quran on physical and mental health.

**Methods:** This review was performed on articles published from the inception and April 2021. Databases including ProQuest, PubMed and Web of Science were searched on 19 April 2021. Keywords such as “Quran”, “al- Quran,” “al- Kareem,” “Holy Quran,” “memori,” “Tahfiz,” “Huffaz,” “listening” and “reciting” were used for databases searching. The risk of bias was assessed using Cochrane risk of bias tool and Joanna Briggs Institute (JBI). Only 20 articles were included in data synthesis out of a total of 230.

**Results:** The findings revealed that listening to, reciting or memorizing the Quran had a favorable effect on depression, anxiety, physiologic parameters, quality of life, quality of sleep and intelligence quotient.

**Conclusion:** The current evidence suggests that, listening to, reciting or memorizing the Quran may be useful as an intervention to improve physical and mental health.

## Introduction

Quran is the Holy book for Muslims. Listening, reciting, and memorizing are activities closely associated with Quran. These activities are particularly akin to listening, singing, and memorizing a song or music. Indulging in music improves individual mood by the release of endorphins via the stimulation of alpha brain waves [1]. Thus, it raises the stress threshold, eliminates negative emotions, and induces a sense of relaxation [2]. It is postulated that listening, reciting, and memorizing Quran may offer similar benefits. Other benefit includes activating and enhancing memory capacity as well as ensuring mental health [3].

Quran memorization is important for practicing Muslims. Memorizing necessitates mental fortitude on the part of the person doing it. The practise of memorizing something learned and sharpening memory through memorization is the most effective way to maintain memory sharpness and brain intelligence [4]. The most common memorization technique is repeated pronunciation or rote learning. This repetition technique increases the brain’s ability to form and retain memories as time passes and the number of sentences memorized increases. The greater the amount of memorizing activity, the greater the brain’s ability to process, remember information and build memory. The body’s neurochemical system is secreted, and the body’s immunity is increased as a result of song appreciation. Aside from that, stress decreases by lowering levels of cortical secreting hormones and cortisol [5].

Memorization is a learning process that involves memory formation, memory storage, memory access and memory reflecting behaviors. Memory is divided into three stages: registration, storage and recall [6]. The human brain is a complex organ. It entails billions of physiological and chemical interactions that result in an experimentally observable neuroelectric activation known as an electroencephalogram (EEG) [7].

Religious practices such as memorizing the Quran are thought to be associated with physical and mental health [8], suggesting it will eventually affect the quality of life (QoL). It leads us to the question whether there is any effect of listening to or reciting or memorizing Quran towards physical and mental health. Therefore, the aim of this systematic review is to evaluate the impact of listening to, reciting or memorizing the Quran on physical and mental health.

## Methods

In this systematic review, databases including ProQuest, PubMed and Web of Science were searched on 19 April 2021. Keywords such as “Quran,” “Qoran,” “al- Quran,” “Koran,” “al- Kareem,” “Holy Quran,” “memori*,” “Tahfidz,” “Tahfiz,” “Huffaz,” “Hafiz,” “listening,” “reciting,” “reading” and Boolean operators such as “AND” and “OR” were used for English databases searching. Searches using the keywords related to the health outcomes (quality of life, physical health, and mental health) have been initially included but resulted in a very limited number of hits. The reviewer team then unanimously decided to exclude the keywords during subsequent search. These resulted in articles that we thoroughly screened for the ones with the intended outcomes (quality of life, physical health, and mental health). All articles that were published between inception and April 2021 were retrieved. The protocol of this systematic review was registered in the International Prospective Register of Systematic Reviews (PROSPERO) with ID number CRD42021258954.

Randomized control trials (RCT), quasi-experimental studies, prospective and retrospective studies and observational studies that assessed the effect of listening to, reciting, or memorizing the Quran on physical and mental health were all included if they met the inclusion criteria. This review included all studies, including those on healthy populations, chronic diseases, and intensive care. There was no gender restriction, but only subjects over the age of 13 were considered. Animal studies, reviews, editorials, letters to the editor, news, and conferences were not included.

The Rayyan-Intelligent Systematic Review was used for the screening. Two independent reviewers determined the studies’ eligibility based on the inclusion criteria. The studies were independently screened by identifying the title and abstract. Following that, all relevant studies must be confirmed in order for the full text to be obtained. If the full text of the articles could not be obtained, we requested them from ResearchGate or contacted the authors and co-authors *via* email. When there were disagreements, another reviewer was added to go over the articles and discuss the resolution. The information and data such as author(s), year of publication, setting, sample size, populations, study designs, methodology, outcome measures and key findings were extracted from articles that met the inclusion criteria.

For RCT, the risk of bias for the included articles was assessed using the Cochrane risk of bias tool [9]. The assessment focused on seven domains: random sequence generation, allocation concealment, blinding of participants and personnel, blinding of outcome assessment, incomplete outcome data, selective reporting, and other sources of bias. Each article was classified as having “low risk of bias,” “unclear/no information” or “high risk of bias.” Meanwhile, critical appraisal tools from Joanna Briggs Institute (JBI) systematic reviews for risk assessment was used in quasi-experimental studies and observational studies. Each article was classified as “yes,” “no,” “unclear” or “not applicable” depending on the questions listed in the checklist for quasi-experimental studies [10] and observational studies [11]. Any disagreements or discrepancies between the two reviewers were resolved by a discussion.

## Results

**FIGURE 1:**
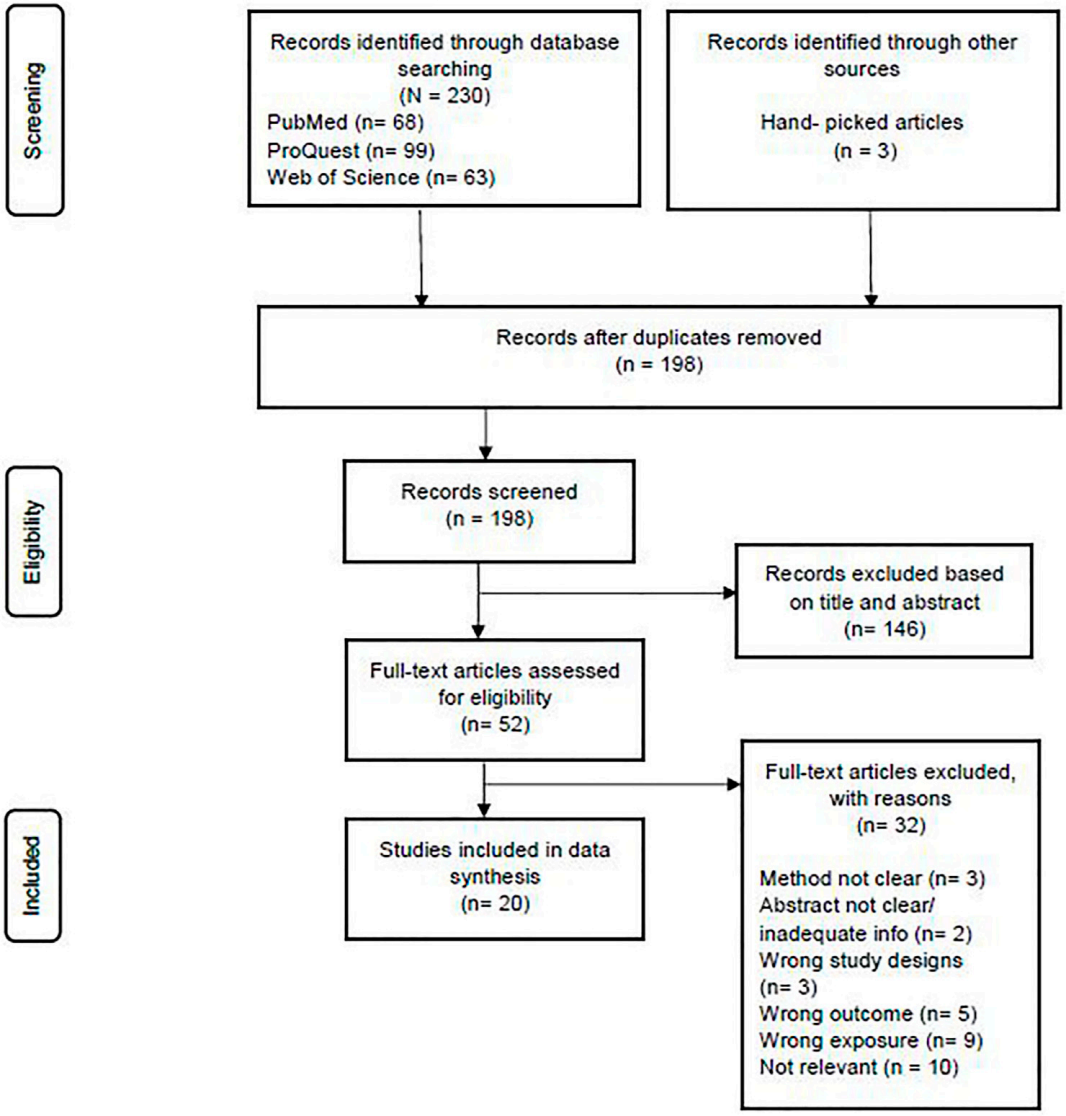
Preferred reporting items for Systematic Reviews and Meta-Analyses (PRISMA) flow diagram (Kuala Lumpur, Malaysia, 2022).

A total of 230 studies were found by searching databases on the initial search query, while 3 studies were found by hand-picking from other sources. Duplicates were removed in 32 cases, and 198 were screened. A total of 146 studies were excluded based on their title and abstract. Meanwhile, 52 studies were evaluated for eligibility based on their full text. Only 20 of these were included for data synthesis, while the remaining 32 were excluded for reasons stated in the Preferred Reporting Items for Systematic Reviews and Meta-Analyses (PRISMA) flow chart (Figure 1).

### Study Characteristics

This systematic review involved 2,566 participants for articles published between inceptions and April 2021. The study designs were RCT or experimental studies (*n* = 7) [5, 12–16, 26], quasi-experimental studies (*n* = 5) [17–21] and observational studies (*n* = 8) [8, 22–26, 28, 29] (Table1).

**TABLE 1:** Study characteristics (Kuala Lumpur, Malaysia, 2022).

Author(s) and publication year	Study designs	Subjects and number of subjects	Intervention/exposure
[15]	Experimental study	Muslim women with depression (*n* = 12)	Experimental group listened to Surah al-Rahman
			Control group exposed to music used for relaxation training
[14]	Experimental study	Older adults (*n* = 65)	Experimental group listened to the Holy Quran for 20 min before sleeping for 4 weeks
			Control group received no intervention
[5]	Experimental study	Healthy males (*n* = 10)	Subjects were asked to recite the Quran and read the book
[27]	Experimental study	Healthy subjects (*n* = 6)	Subjects were instructed to rest and listen to music and Quran recitation for 3 min using headphones with their eyes closed
[16]	RCT	ICU patients (*n* = 55)	Experimental group received 30 min of Holy Quran recitation (HQR)
			Control group had 30 min of rest in bed before the start of the weaning of mechanical ventilation
[13]	RCT	Haemodialysis patients (*n* = 54)	Experimental group listened to the recitation of the Holy Quran
			Control group received no intervention
[12]	RCT	Hemodialysis Patients (*n* = 60)	Experimental group listened to the Quran recitation in a traditional cantillation voice
			Control group received no intervention
[20]	Quasi-experimental	Mental health personnel (*n* = 81)	Experimental group listened to the Quran Tartil for 15 min every morning for 2 months
			Control group received no intervention
[18]	Quasi-experimental	Coronary heart diseases patients (*n* = 80)	Experimental group listened to the Quran recital five times for 24 h
			Control group received routine care
[21]	Quasi-experimental	Elderly (*n* = 60)	Experimental group attended 36 sessions of listening to Qur’anic recital and 3 sessions of sermon by a preacher
			Control group received treatment as usual
[17]	Quasi-experimental	Pregnant women (*n* = 40)	Experimental group listened to ar-Rahman surah at the time of labor
			Control group received no intervention
[19]	Quasi-experimental	ICU patients (*n* = 30)	Experimental group listened to Yousef Surah
			No control group
[23]	Observational study	Hafiz Non-Hafiz students (*n* = 32)	Three groups
			Memorized Quran
			Not memorized Quran but familiar with Quran
			Not memorized Quran and not familiar with Quran
[8]	Observational study	Older men (*n* = 400)	Five memorization categories
			<0.5 sections
			0.5–1 sections
			2–4 sections
			5–9 sections
			10–30 sections
[28]	Observational study	High schools students (*n* = 956)	Case group
			Control group
[29]	Observational study	Healthy adults (*n* = 63)	Three groups
			Completely memorized Quran (CMQ)
			Partially memorized Quran (PMQ)
			Control (CON)
[25]	Observational study	Palliative radiotherapy patients (*n* = 90)	All subjects were listened, read and watched the text of the Holy Quran
[22]	Observational study	Tahfiz students (*n* = 116)	Subjects were divided into
			Level 1 (1–10 chapters)
			Level 2 (11–20 chapters)
			Level 3 (21–30 chapters)
[24]	Observational study	Tahfiz students (*n* = 105)	Subjects were divided into
			Level 1 (1–10 chapters)
			Level 2 (11–20 chapters)
			Level 3 (21–30 chapters)
[26]	Observational study	Students (*n* = 131)	Subjects comprised of Tahfiz and non- Tahfiz students

Thirteen studies assessed the effect of listening al- Quran on depression [13, 15, 18], mental health [20], quality of sleep [14], physiological parameters [8, 16, 19], anxiety [12, 17], QoL [21, 25] and brain activity [27]. One study assessed the effect of reciting the al- Quran on behaviors of mind [5]. Meanwhile, seven studies assessed the effect of memorizing al- Quran on memory capacity [23], health status [8] depression [28], brain tissue [29], QoL [22] and intelligent quotient (IQ) [24, 26].

### Outcome Measures

Beck Depression Inventory-II (BDI-II) [13, 15, 28], standard health questionnaire [8, 20], Pittsburgh sleep quality index [14], physiological parameters [16, 19], Spielberger’s State-Trait Anxiety Inventory (STAI) [12], depression, anxiety and stress score (DASS) [18], World Health Organization QoL (WHOQoL) [21], Wechsler Memory Scale III [23], magnetic resonance imaging (MRI) [29], electroencephalogram (EEG) [5, 27], European Organization for Research and Treatment of Cancer C30 Scale Quality of Life Questionnaire (EORTC C30 Scale QLQ) [25], Short Form-36 QoL (SF-36) [22] and Wechsler Abbreviated Scale of Intelligence II (WASI II) [24, 26] were used as the outcome measures for each study. Details on the assessments and outcome measures are presented in Table 2.

**TABLE 2:** Outcome measures (Kuala Lumpur, Malaysia, 2022).

Author(s) and publication year	Assessment	Outcomes
[15]	Depression in both groups were assessed by BDI- II	Depression scores on pre- assessment did not differ significantly between groups (*p* = 0.75)
		Both groups had decreased levels of depression on post- assessment (*p* < 0.05)
		There was a significant difference in depression reduction between the control (*p* < 0.05) and experimental (*p* < 0.05) groups
		The experimental group decreased significantly more than the control group (*p* < 0.05)
[20]	Mental health was assessed by a standard mental health questionnaire (12 items)	There were significant changes in scores in the experimental group (*p* < 0.001) but not in the control group (*p* = 0.70) before and after the intervention
		There were no significant differences in mean mental health scores between experimental and control groups before intervention (*p* = 0.15), but significant differences after intervention (*p* < 0.05)
[14]	Pittsburgh sleep quality index (PSQI)	There was a significant difference between the experimental and control groups after listening to the Holy Quran (*p* < 0.001)
		The two groups differed significantly in terms of habitual sleep efficiency (*p* < 0.001), daytime dysfunction (*p* > 0.05) and total sleep quality (*p* < 0.001) at the post- intervention
[16]	Physiological parameters (rapid shallow breathing index, respiratory rate (RR), heart rate (HR), oxygen saturation SpO_2_, exhaled carbon dioxide and blood pressure (BP))	There was no significant difference in all parameters between the groups; RR (*p* = 0.50), HR (*p* = 0.20), BP and SpO2 (0.07)
		There were no significant differences in patients’ recovery using either the conventional weaning method or HQR during weaning
[13]	Depression in both groups were assessed by BDI- II	BDI-II scores in the experimental group decreased from 33.6 to 14.5 compared to a slight increase in the BDI-II from 29.3 to 31.6 in control group (*p* < 0.0001)
[12]	Anxiety in both groups was assessed by STAI	The experimental group experienced a reduction in overall anxiety score of 46.4 points, compared to an increase of 1.8 points in the control group
		There was a significant difference in overall STAI scores between experimental and control groups at baseline and on follow- up (*p* < 0.0001)
		The experimental group experienced a significant decline in anxiety scores on the STAI compared to the control group (*p* < 0.001)
[18]	Depression, Anxiety and Stress Score (DASS)	There was an improvement from baseline to follow-up in the DASS score in both groups
		Mean differences of all DASS scores were higher in the experimental group, with a statistically significant difference in improving stress score favoring intervention (*p* < 0.001)
[21]	Depression was assessed by Geriatric Depression Scale (GDS)	There were statistically significant reductions in depression scores after the 12-week intervention (*p* < 0.001)
	QoL was assessed by WHOQoL Indonesian version	There was a statistically significant difference in GDS scores between groups at the 4th, 8th, and 12th week post-intervention (*p* < 0.001)
		There was a statistically significant improvement in QoL mean scores in both intervention and control groups at the 12-week post-intervention (*p* < 0.001)
		There was statistically significant difference in QoL mean scores between intervention and control groups at week 12 post-intervention (*p* < 0.001)
[17]	The clear tool used to measure anxiety was not mentioned in the article	There was significant reduction in the level of anxiety between pre- and post- intervention for the experimental group (*p* < 0.01) but no significant difference for the control group (*p* = 0.50)
	Cortisol level was measured	There was a significant difference of mean rank between experimental and control groups in terms of anxiety score changes (*p* < 0.001), cortisol level (*p* < 0.05), and time of labor (*p* < 0.001)
[19]	Physiological parameters were measured (BP, RR, PR and consciousness level)	Significant differences were observed (*p* < 0.0001) in that the rate of vital signs declined after intervention
		There was a significant difference between the level of consciousness before and after the intervention (*p* < 0.0001), increasing the level of consciousness after the intervention
[23]	Wechsler Memory Scale III	No differences in List I total words correctly recalled score (*p* = 0.359), List II Recognition Score (*p* = 0.537), Short Delay Recall performance (*p* = 0.603) or Long Delay Recall (*p* = 0.666)
[8]	Depression was assessed by a standardized questionnaire	Hypertension, diabetes, and depression decreased significantly across the increased categories of memorization (*p* < 0.0001)
	BP was measured using a sphygmomanometer	
	Glucose was measured using glucometer (Accu- Check Active)	
[28]	Depression was assessed by BDI- II	When scores of attitude and depression scales are compared with each other in terms of demographic parameters, there is a difference among group, gender and age parameters (*p* < 0.001)
		There was a weak positive significant correlation between attitude scale and BDI- II for case and control (*p* < 0.001)
[29]	Brain atrophy was measured using MRI	There were significant differences among groups; Gray Matter (GM) (*p* < 0.05), White Matter (WM) (*p* < 0.05), Cerebrospinal Fluid (CSF) (*p* < 0.05), Intracranial Volume (ICV) (*p* < 0.001), Total Brain Volume (TBV) (*p* < 0.001) GM for CMQ > CON (*p* < 0.001) GM for PMQ > CON (*p* < 0.001) There is no diff. between CMQ and PMQ (*p* = 0.108) WM for CMQ > CON (*p* < 0.001) CSF for CMQ > CON (*p* < 0.001) ICV for CMQ vs. PMQ vs. CON (*p* < 0.001) TBV for CMQ vs. PMQ vs. CON (*p* < 0.001)
[25]	QoL was assessed by EORTC C30 Scale QLQ	There was a significant difference for frequency and duration of Quran recitation among patients before and after their cancer diagnosis (*p* < 0.05)
		There was a correlation between Quran recitation and subjective well-being (*p* < 0.001). There was a correlation between Quran recitation and increasing life expectancy (*p* < 0.05)
[5]	Brainwave was measured using EEG	The power spectral densities (PSD) were higher during reciting the Quran than before the recitation
		There was a negative correlation between reading books and reciting the Quran for each subject
[27]	Relaxation of mind was measured using EEG	The brain activity is less active and the Alpha Power is higher when the subject is listening to Quran recitation
[22]	QoL was measured using Short Form-36 QoL (SF-36) Bahasa Malaysia version	A significant positive relationship between al-Quran memorization with physical health (*r* = 0.300, *p* < 0.05) and mental health (*r* = 0.194, *p* < 0.05). There were significant differences (*p* < 0.05) for physical health, physical function dimension, general health dimension and social function dimension
[24]	IQ was evaluated by WASI- II	There was a weak positive correlation between the level of IQ with the level of memorization of the Quran (*r* = 0.375, *p* < 0.001). There was a significant relationship between the level of IQ with the level of memorization of the Quran
[26]	IQ was evaluated by WASI- II	There was a moderate positive correlation between IQ and the level of memorization (*r* = 0.375, *p* < 0.001)

### Risk of Bias

**FIGURE 2:**
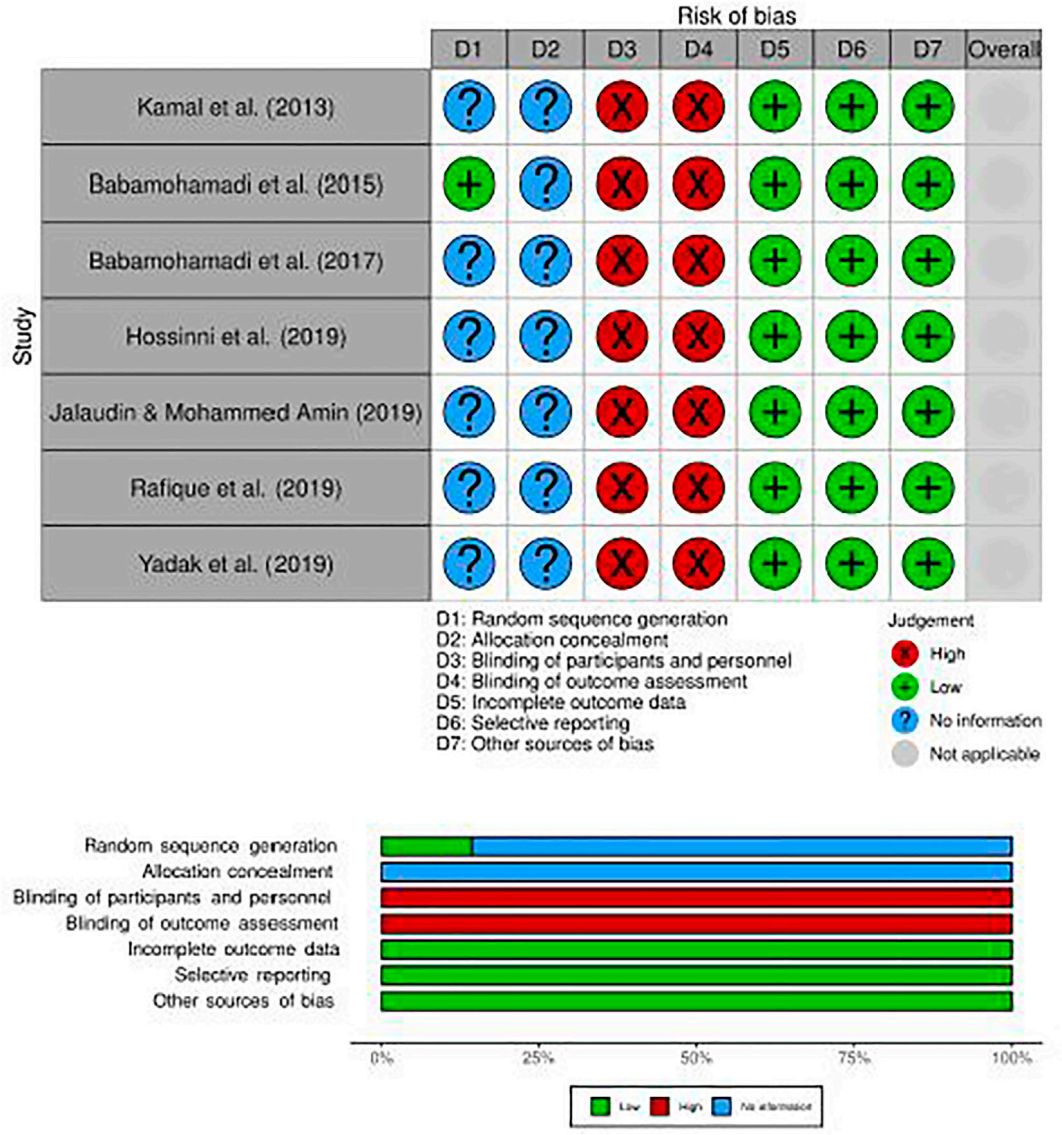
A plotted graph and summary of risk of bias for RCT studies (Kuala Lumpur, Malaysia, 2022).

Only one study described the participants’ randomization process using a coin toss [12], however, the other studies did not describe the randomization process. There was no clear information on the method of allocation concealment in any of the studies. All studies failed to provide information on the blinding of participants, personnel, and outcome assessment. All studies had a low risk of bias for incomplete outcome data because there were no missing data, and the intervention groups were evenly distributed. All studies were found to be free of biased reporting data and other potential sources of bias. Figure 2 depicts a plotted graph and summary of risk of bias of RCT and experimental studies.

According to the Joanna Briggs Institute (JBI) critical checklist for quasi-experimental studies, all studies clearly addressed aspects of ambiguity regarding the “cause” was manipulated before the occurrence of the “effect.” Besides, no differences between participants in the compared groups, no other differences between groups in terms of treatments or care received, and there were independent, separate groups used as control groups (except for [19] there was no control group involved). Moreover, there were multiple measurements of the outcome for both pre- and post-intervention, completed follow up, the outcomes of participants included in any comparisons were measured in the same and reliable way and appropriate statistical analysis was used (Table 3).

**TABLE 3:** Joanna Briggs Institute (JBI) critical appraisal checklist for quasi-experimental studies (Kuala Lumpur, Malaysia, 2022).

Author(s) and publication year	Q1	Q2	Q3	Q4	Q5	Q6	Q7	Q8	Q9
	Is it clear in the study what is the “cause” and what is the “effect” (i.e., there is no confusion about which variable comes first)?	Were the participants included in any comparisons similar?	Were the participants included in any comparisons receiving similar treatment/care, other than the exposure or intervention of interest?	Was there a control group?	Were there multiple measurements of the outcome both pre and post the intervention/exposure?	Was follow up complete and if not, were differences between groups in terms of their follow up adequately described and analyzed?	Were the outcomes of participants included in any comparisons measured in the same way?	Were outcomes measured in a reliable way?	Was appropriate statistical analysis used?
[17]	Yes	Yes	Yes	Yes	Yes	Yes	Yes	Yes	Yes
[18]	Yes	Yes	Yes	Yes	Yes	Yes	Yes	Yes	Yes
[21]	Yes	Yes	Yes	Yes	Yes	Yes	Yes	Yes	Yes
[20]	Yes	Yes	Yes	Yes	Yes	Yes	Yes	Yes	Yes
[19]	Yes	Yes	Yes	No	Yes	Yes	Yes	Yes	Yes

For JBI critical checklist for observational studies, six studies clearly defined the inclusion criteria [8, 22–24, 28, 29], whereas the other two studies were unclear [26] and no inclusion criteria mentioned [25]. All studies described the subjects and settings in detail, the exposures were measured in a valid and reliable manner, and the condition was measured using standard criteria. Confounding factors were identified and strategies for dealing with confounding were clearly stated in two studies [22, 26]. As far as we could tell, all the studies measured the outcomes in a valid and reliable manner, and appropriate statistical analysis was used (Table 4).

**TABLE 4:** JBI critical appraisal checklist for observational studies (Kuala Lumpur, Malaysia, 2022).

Author(s) and publication year	Q1	Q2	Q3	Q4	Q5	Q6	Q7	Q8
	Were the criteria for inclusion in the sample clearly defined?	Were the study subjects and the setting described in detail?	Was the exposure measured in a valid and reliable way?	Were objective, standard criteria used for measurement of the condition?	Were confounding factors identified?	Were strategies to deal with confounding factors stated?	Were the outcomes measured in a valid and reliable way?	Was appropriate statistical analysis used?
[22]	Yes	Yes	Yes	Yes	Yes	Yes	Yes	Yes
[23]	Yes	Yes	Yes	Yes	No	No	Yes	Yes
[24]	Yes	Yes	Yes	Yes	No	No	Yes	Yes
[25]	No	Yes	Yes	Yes	No	No	Yes	Yes
[26]	Unclear	Yes	Yes	Yes	Yes	Yes	Yes	Yes
[28]	Yes	Yes	Yes	Yes	No	No	Yes	Yes
[29]	Yes	Yes	Yes	Yes	No	No	Yes	Yes
[8]	Yes	Yes	Yes	Yes	No	No	Yes	Yes

## Discussion

This systematic review is aimed to assess the effect of listening to, reading, or memorizing al- Quran on physical and mental health. Spirituality affects human health [30, 31]. Spiritual approaches have favorable roles in addressing matters related to mental health [32]. It is also stated that spirituality influences the quality of life, and it is needed by adolescents so that they have careful guidance, have a brighter future and be prepared for adversity.

The findings of this systematic review reveal that listening to al- Quran especially Surah al- Rahman is helpful in managing depression among group of Muslim women [15] and reduces anxiety burden of pregnant women in facing the labor process [17]. Listening to the Holy Quran recitation also had a significant effect on lowering depressive symptoms in haemodialysis patients [9]. Not only that, listening to the Quran is an effective intervention for reducing anxiety among patients undergoing haemodialysis [12]. Listening to Quran recitation resulted in a better tendency for improvement in depression, anxiety and stress scores among patients diagnosed with chronic heart diseases [18]. Moreover, listening to Yasin Surah is believed to have more psychological impact to produce more calmness and tranquility to mind [33].

Psychologists could recommend Quran listening for improving mental health and achieve greater serenity for mental health personnel [20]. In another study done on the elderly adults, it was found that listening to the voice of Quran recitation could affect sleep quality [14]. Positive effect and improvement in physiological parameters and stress reduction could be seen among ICU patients after listening to Quran recitation [16]. Listening to the Quran could reduce the vital signs and increase the level of consciousness among ICU patients [19].

In terms of quality of life (QoL), religious-based intervention such as listening to the Quran has a greater impact on relieving depression and improving QoL [21]. Listening to the Quran improves QoL and life expectancy in palliative radiotherapy patients [25]. Memorizing the Quran has the potential to improve the QoL as well as mental and physical health among Tahfiz students [22]. The higher the level of Quran memorization, the higher the level of IQ [24, 26].

Memorizing the Quran can improve people’s mental health and be an effective stress reliever. The higher the memorization, the stronger the psychological impulse towards their beliefs, including feelings of happiness, contentment, and positive attitudes. These readings can provide health benefits similar to prayer or singing for people of other faiths. There was a strong linear relationship between Quran memorization and hypertension, diabetes, and depression implying that those who had memorized most of the Quran were less likely to suffer from one of these chronic diseases [8]. Memorizing the Quran may also aid in the treatment of depression. When religious behavior among high school students increases, depression decreases [28]. Findings from Rahman et al. [29] showed that memorizing the Quran could affect brain atrophy. The volume of grey, white, and total brain in those who memorized the Quran was greater than those who memorized only part of the Quran or did not memorize the Quran because the more brain is used, the more likely that the brain tissue is preserved. When a subject listens to Quran recitation, his or her brain activity decreases and his or her Alpha power increases. Hence, this could be a useful tool for a healthy and happy mind assisting people in recognizing the importance of if Islamic practices in their lives [27]. Instead of listening and memorizing, reading or reciting the Quran may result in a state of mind that is restful and calming. Moreover, this Quran recitation could be used as a tool for meditation, as it reduces stress and promotes mental calmness [5].

There are some limitations encountered while conducting this review. This field of study is greatly understudied. In addition, there are many inconclusive findings have been made. Therefore, there are huge gaps in the studied area that we need to discover and address in the future.

### Conclusion

The current evidence suggests that listening to, reciting or memorizing the Quran is a helpful non-invasive tool or intervention for reducing stress, depression, anxiety and improving QoL among Muslims. This can be seen in the effect of these three exposures on physical and mental health, as collated in this systematic review. However, due to the scarcity of relevant studies, additional research is required to obtain more information in this study’s area.

## References

[1] Almerud S Petersson K. Music Therapy - A Complementary Treatment for Mechanically Ventilated Intensive Care Patients. Intensive Crit Care Nurs (2003) 19(1):21–30. 10.1016/S0964-3397(02)00118-0 12590891

[2] Chang M Chen C Huang K. Universality in the Brain while Listening to Music. Proc Biol Sci (2007) 17:2580–433. 10.1098/rspb.2001.1802 PMC1088896 11747560

[3] Hojjati A Rahimi A Farehani MD Sobhi-Gharamaleki N Alian B. Effectiveness of Quran Tune on Memory in Children. Proced - Soc Behav Sci (2014) 114:283–6. 10.1016/j.sbspro.2013.12.699

[4] Chek Y Yaacob M. Kajian tinjauan terhadap penjadualan kelas hafazan di Pusat Pengajian Tahfiz. Kuala Kubu Bharu, Malaysia: Darul Quran JAKIM (2016).

[5] Kamal NF Mahmood NH Zakaria NA. Modeling Brain Activities during Reading Working Memory Task: Comparison between Reciting Quran and Reading Book. Proced - Soc Behav Sci (2013) 97:83–9. 10.1016/j.sbspro.2013.10.207

[6] Schunk D. Learning Theories: An Educational Perspective. London, United Kingdom: Pearson education, Inc. (2012).

[7] Bhattacharya J Petsche H. Universality in the Brain while Listening to Music. Proc Biol Sci (2012) 68(1484):2423338–3. 10.1098/rspb.2001.1802 PMC1088896 11747560

[8] Saquib N Saquib J Alhadlag A Albakour MA Aljumah B Sughayyir M Health Benefits of Quran Memorization for Older Men. SAGE Open Med (2017) 5:1–7. 10.1177/2050312117740990 PMC5686875 29163949

[9] Higgins JPT Savović J Page MJ Elbers RG Sterne JAC. Assessing Risk of Bias in a Randomized Trial. Cochrane Handbook Syst Rev Interventions (2019) 2019:205–28. 10.1002/9781119536604.ch8

[10] Tufanaru C Munn Z Aromataris E Campbell J Hopp L. Chapter 3: Systematic Reviews of Effectiveness. In: Aromataris E Munn Z, editors. JBI Quasi-Experimental Appraisal Tool (2017).

[11] Moola S Munn Z Tufanaru C Aromataris E Sears K Sfetcu R Checklist for Analytical Cross-Sectional Studies. Adelaide, Australia: Joanna Briggs Institute (2020). Available from: https://jbi.global/sites/default/files/2019-05/JBI_Critical_Appraisal-Checklist_for_Analytical_Cross_Sectional_Studies2017_0.pdf (Accessed August 31, 2021).

[12] Babamohamadi H Sotodehasl N Koenig HG Jahani C Ghorbani R. The Effect of Holy Qur'an Recitation on Anxiety in Hemodialysis Patients: A Randomized Clinical Trial. J Relig Health (2015) 54(5):1921–30. 10.1007/s10943-014-9997-x 25559332

[13] Babamohamadi H Sotodehasl N Koenig HG Al Zaben F Jahani C Ghorbani R. The Effect of Holy Qur’an Recitation on Depressive Symptoms in Hemodialysis Patients: A Randomized Clinical Trial. J Relig Health (2017) 56(1):345–54. 10.1007/s10943-016-0281-0 27393704

[14] Hossini A Azimian J Motalebi SA Mohammadi F. The Effect of Holy Quran Recitation on the Quality of Sleep Among Older People Residing in Nursing Homes. Iranian J Ageing (2019) 14(2):236–47. 10.32598/sija.13.10.280

[15] Rafique R Anjum A Raheem SS. Efficacy of Surah Al-Rehman in Managing Depression in Muslim Women. J Relig Health (2019) 58(2):516–26. 10.1007/s10943-017-0492-z 28900859

[16] Yadak M Ansari KA Qutub H Al-Otaibi H Al-Omar O Al-Onizi N The Effect of Listening to Holy Quran Recitation on Weaning Patients Receiving Mechanical Ventilation in the Intensive Care Unit: A Pilot Study. J Relig Health (2019) 58(1):64–73. 10.1007/s10943-017-0500-3 28965157

[17] Irmawati HV Syamsuddin S Arundhana AI. The Effect of Listening to the Recitation of Qur’an (Murottal Ar-Rahman Surah) on the Level of Anxiety of Pregnant Women in Siti Fatimah Maternal and Child Hospital. Enferm Clin (2020) 30:238–42. 10.1016/j.enfcli.2019.07.097

[18] Jayus R Sharif Abdullah SS Letchumy S Che Hassan HH Choor CK Mohamad MSF The Effect of Listening to the Quran Recital on Depression, Anxiety and Stress Among Coronary Heart Disease Patients. Int J Cardiol (2017) 249:S41. 10.1016/j.ijcard.2017.09.145

[19] Nasiri AA Shahdadi H Mansouri A. An Investigation into the Effect of Listening to the Voice of the Holy Quran on Vital Signs and Consciousness Level of Patients Admitted to the ICU Wards of Zabol University of Medical Sciences Hospitals. World Fam Med Journal/Middle East J Fam Med (2017) 15(10):75–9. 10.5742/mewfm.2017.93142

[20] Mahjoob M Nejati J Hosseini A Bakhshani NM. The Effect of Holy Quran Voice on Mental Health. J Relig Health (2016) 55(1):38–42. 10.1007/s10943-014-9821-7 24421119

[21] Pramesona BA Taneepanichskul S. The Effect of Religious Intervention on Depressive Symptoms and Quality of Life Among Indonesian Elderly in Nursing Homes: A Quasi-Experimental Study. Clin Interv Aging (2018) 13:473–83. 10.2147/CIA.S162946 29606860 PMC5868573

[22] Abd Rahman S Ishak I Abd Warif N Ibrahim F Che Din N Harun D Hubungan antara hafazan al- Quran dan kualiti hidup pelajar Tahfiz di Selangor, Malaysia. Jurnal Sains Kesihatan Malaysia (2019) 17(SI):11–1111. 10.17576/jskm-2019-01

[23] Black R Mushtaq F Baddeley A Kapur N. Does Learning the Qur’an Improve Memory Capacity? Practical and Theoretical Implications. Memory (2020) 28(8):1014–23. 10.1080/09658211.2020.1811347 32870071

[24] Ghazali A Mohamad A Ibrahim F Che Din N Abd Warif N Harun D Determination of Level of Heavy Metals, Al-Quran Memorization and Intelligence Quotient (IQ) Among Tahfiz Students in Selangor. Tahfiz di Selangor (2019) 17(2):135–46. 10.17576/jskm-2019-1702-16

[25] Hematti S Baradaran-Ghahfarokhi M Khajooei-Fard R Mohammadi-Bertiani Z. Spiritual Well-Being for Increasing Life Expectancy in Palliative Radiotherapy Patients: A Questionnaire-Based Study. J Relig Health (2015) 54(5):1563–72. 10.1007/s10943-014-9872-9 24797156

[26] Ibrahim FW Abdul Rahman NF Abd Rahman S Abd Warif NM Harun D Ghazali AR Dietary Intake, Levels of Trace Elements and Intelligence Quotient (IQ) Among Huffaz Students from Selected Tahfiz Schools in Selangor. Jurnal Sains Kesihatan Malaysia (2018) 16:129–36. 10.17576/jskm-2018-16si-18

[27] Jalaudin N Mohammed Amin MK. EEG Analysis on Human Reflection towards Relaxation of Mind. Mal J Fund Appl Sci (2019) 15(2):185–9. 10.11113/mjfas.v15n2.1103

[28] Ozturk O Celik AM Uyar EI. The Relation of Religious Attitudes and Behaviours with Depression in Boarding Quran Course Students. Psychiatr Danub (2016) 28(4):379–85. 27855429

[29] Rahman MA Aribisala BS Ullah I Omer H. Association between Scripture Memorization and Brain Atrophy Using Magnetic Resonance Imaging. Acta Neurobiol Exp (Wars) (2020) 80(1):90–7. 10.21307/ane-2020-009 32214278

[30] Bonelli RM Koenig HG. Mental Disorders, Religion and Spirituality 1990 to 2010: A Systematic Evidence-Based Review. J Relig Health (2013) 52(2):657–73. 10.1007/s10943-013-9691-4 23420279

[31] Leondari A Gialamas V. Religiosity and Psychological Well-Being. Int J Psychol (2009) 44(4):241–8. 10.1080/00207590701700529 22029552

[32] Karimipour M Sawari SSM Hafiz MMA. Religion, Spirituality and Mental Health: A Review of Literature. Res J Commerce Behav Sci (2015) 5(1):42–7.

[33] Alhouseini AMRA Al-Shaikhli IF Rahman AWBA Alarabi K Dzulkifli MA. Stress Assessment while Listening to Quran Recitation. In: Proceedings - 2014 International Conference on Computer Assisted System in Health; 19-21 December 2014; Kuala Lumpur, Malaysia (2014). p. 67–72. 10.1109/CASH.2014.14

